# MyThisYourThat for interpretable identification of systematic bias in federated learning for biomedical images

**DOI:** 10.1038/s41746-024-01226-1

**Published:** 2024-09-07

**Authors:** Klavdiia Naumova, Arnout Devos, Sai Praneeth Karimireddy, Martin Jaggi, Mary-Anne Hartley

**Affiliations:** 1 Laboratory for Intelligent Global Health and Humanitarian Response Technologies (LiGHT), Machine Learning and Optimization Laboratory, Swiss Federal Institute of Technology Lausanne (EPFL), Lausanne, Switzerland; 2 ETH AI Center, Swiss Federal Institute of Technology Zurich (ETH Zurich), Zurich, Switzerland; 3 Berkeley AI Research Laboratory, University of California, Berkeley, CA USA; 4 Department of Computer Science, University of Southern California, Los Angeles, CA USA; 5 Machine Learning and Optimization Laboratory, Swiss Federal Institute of Technology Lausanne (EPFL), Lausanne, Switzerland; 6 Laboratory for Intelligent Global Health and Humanitarian Response Technologies (LiGHT), School of Medicine, Yale University, New Haven, CT USA

**Keywords:** Computer science, Computational science, Medical imaging, Health care, Biomedical engineering

## Abstract

Distributed collaborative learning is a promising approach for building predictive models for privacy-sensitive biomedical images. Here, several data owners (clients) train a joint model without sharing their original data. However, concealed systematic biases can compromise model performance and fairness. This study presents MyThisYourThat (MyTH) approach, which adapts an interpretable prototypical part learning network to a distributed setting, enabling each client to visualize feature differences learned by others on their own image: comparing one client’s 'This’ with others’ 'That’. Our setting demonstrates four clients collaboratively training two diagnostic classifiers on a benchmark X-ray dataset. Without data bias, the global model reaches 74.14% balanced accuracy for cardiomegaly and 74.08% for pleural effusion. We show that with systematic visual bias in one client, the performance of global models drops to near-random. We demonstrate how differences between local and global prototypes reveal biases and allow their visualization on each client’s data without compromising privacy.

## Introduction

The transformative force of deep learning on clinical decision-making systems is being increasingly documented^1–3^. For medical images, these advances have the potential to improve and democratize access to high-quality standardized interpretation, extracting predictive features at a granularity previously inaccessible to human experts who are often lacking in low-resource settings. The potential to automate routine analysis of medical records^3^ and help find hidden predictive patterns in the data that may reduce errors and unnecessary interventions (for example, biopsy)^4^ moves us towards more efficient, personalized, and accessible healthcare.

However, the performance of these models relies on large, carefully curated centralized databanks, which are often challenging to create or access in practice. Rather, medical data are usually fragmented among several institutions that are unable to share due to a range of well-considered reasons. DIStributed COllaborative (DISCO) learning has emerged as a solution to this issue, offering privacy-preserving collaborative model training without sharing any original data. Here, instead of sending the data to a central model, the model itself is distributed to the data owners to learn in situ, updating a global model via privacy-preserving gradients. When a central server is used, the technique is known as federated learning (FL)^5^, and it has already been shown to hold potential for various medical applications^6–9^.

While DISCO addresses the issue of data privacy, it comes at a cost to transparency, resulting in clients learning blindly from their peers. In this *black-box data* setting, hidden biases between clients of the federation can make generalization challenging, and even when there are no biases present, its risk may degrade trust. Coupled with the already poor transparency of deep learning architectures used for medical images (aka black-box models), interpretability is becoming a critical feature to ensure a balance in the trade-off between transparency and privacy that will encourage implementation.

Specifically required, is an approach to inspect data *interoperability* between clients as well as provide insights into the most predictive features. Additionally, this approach should be adept at detecting and quantifying concealed biases in the data in an interpretable way, while preserving clients’ privacy. For instance, in shortcut-learning, a model uses a proxy feature, which is systematically associated with a label to predict that label (e.g. using the hospital logo on an X-ray to diagnose tuberculosis because this infection is specifically treated in that hospital).

As for the black-box neural networks, numerous approaches attempt to *explain* them by a posthoc analysis of their predictions^10–16^. Many of these methods have been well summarized elsewhere^17,18^. Feature visualization (https://distill.pub/2017/feature-visualization/) and saliency mapping with Grad-CAM^12^ are among the most popular techniques. These methods visualize the regions in data that are most determinant for the prediction, however, they fail to tell us why or to what extent the visualized regions are essential for a prediction^19^.

This work aims to adapt an inherently interpretable model (IM) to the FL setting^20,21^. Many IMs exist for tabular data, for example, sparse logical models such as decision trees and scoring systems. For image recognition tasks, models that possess human-friendly reasoning based on a similarity between a test instance and already known instances (e.g. nearest neighbors from a training set or the closest samples from a set of learned prototypes) are particularly promising. This learning approach opens possibilities for incorporating the scrutiny of domain experts, allowing them to debug the model’s logic and examine the quality of training data. Prototypical part learning neural network (ProtoPNet) developed by Chen et al.^22^ is a popular IM for images and is the method we adopt for FL in this work.

To summarize, ProtoPNet (Supplementary Fig. 1) uses a set of convolutional layers to map input images to a latent space, followed by a prototype layer, which learns a set of prototypical parts from encoded training images that best represent each class. Classification then relies on a similarity score computed between these learned prototypes and an encoded test image. A prototype can be visualized by highlighting a patch in an input image, which is the closest in terms of squared *L*_2_-distance to this prototype in a latent space. The performance of ProtoPNet was demonstrated on the task of bird species identification. The model showed an accuracy comparable with the state-of-the-art black-box deep neural networks while being easily interpretable. ProtoPNet was further extended to perform the classification of mass lesions in digital mammography^23^ and image recognition with hierarchical prototypes^24^.

**Fig. 1:**
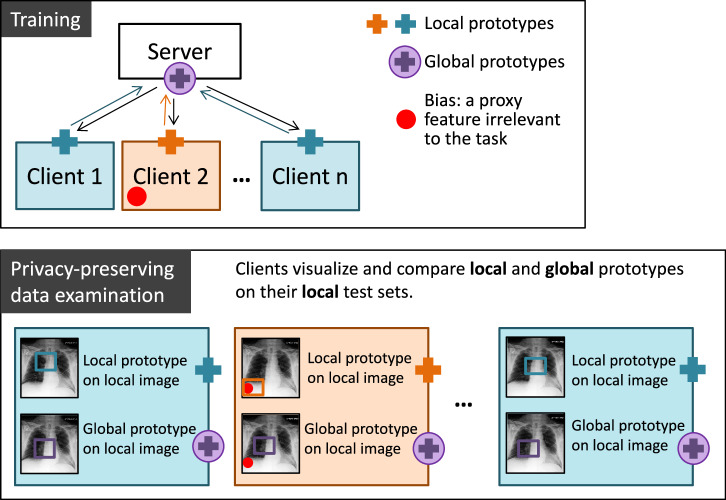
Schematic representation of the MyTH approach. Within one communication round, each client learns local prototypes (blue and orange crosses) on its local training set and shares them with a server that aggregates and averages local prototypes from all clients and sends these new global prototypes (circled purple cross) back to clients. After several communication rounds, each client can examine the global data locally by visualizing and comparing local and global prototypes on its private local test set. Possible hidden bias in the federation will result in a large difference between local and global prototypes.

In this work, we develop an approach called MyTH (MyThisYourThat) through the adaptation of ProtoPNet to FL and demonstrate its capacity for identifying bias in medical images. The idea is that prototypes learned on each client’s local data represent feature importance from that client’s *point of view*. As summarized in Fig. 1, clients learn local prototypes separately and send them to a server that aggregates and averages local prototypes to obtain global ones and sends them back to clients. The patches most activated by each of these two types of prototypes can be visualized and compared on each client’s local test set without a need to share the data. By comparing global and local prototypes, the clients can assess the interoperability of the data and directly examine the predictive impact of other clients without compromising their privacy. i.e. compare one client’s ’This’ with others ’That’. To the best of our knowledge, this work is the first attempt at creating an interpretable methodology to inspect the interoperability of biomedical imaging data in FL.

Our main contributions are as follows:

1. We introduce MyTH adapting ProtoPNet to a federated setting to enable privacy-preserving identification of visual data bias in FL.
2. We formalize a set of use cases for interpretable distributed learning on imperfectly interoperable biomedical image data containing hidden bias.
3. We demonstrate the performance of our MyTH approach on a benchmark dataset of human X-rays and compare it to baseline models.
4. We show how MyTH helps identify biased clients in FL without disclosing the data.
5. Finally, we propose a new approach to use MyTH for interpretable personalization.

## Results

### Quantitative results

We experimented on the CheXpert dataset^25^, a large public dataset of chest X-rays, after processing it to allow for binary classification of cardiomegaly and pleural effusion conditions (see the “Methods” section).

#### Unbiased setting

For both tasks, we first trained a Centralized Model (CM), i.e. ProtoPNet baseline. We then partitioned the data in an independently and identically distributed (IID) manner over four clients and trained Local models (LM) on each client’s data without collaboration. After that, clients collaboratively trained Global (GM) and Personalized (PM) models sharing either all (GM) or part (PM) of their networks parameters.

The balanced accuracy for these four types of models trained on unbiased data is presented in Table 1. The CM gives 74.45% and 75.95% balanced accuracy for cardiomegaly and pleural effusion classification, respectively. As expected, LMs perform worse than centralized ones due to the smaller dataset: LMs achieve 71.64% for cardiomegaly and 70.66% for pleural effusion classes. When the clients train ProtoPNet in collaboration, its performance improves: GM achieves 74.14% balanced accuracy for cardiomegaly and 74.08% for pleural effusion classes, which are close to the values achieved by the corresponding CM. Personalized models, however, demonstrate worse performance of 63.74% and 63.76% for cardiomegaly and pleural effusion classes, respectively, which may be the consequence of exchanging only a part of the network: prototypes and weights of the final layer. The values of classification sensitivity and specificity used to compute balanced accuracy can be found in Supplementary Table 1.

**Table 1:** Centralized vs federated unbiased settings

Model	CM	LM	GM	PM
Cardiomegaly	74.45 ± 0.73	71.64 ± 1.05	74.14 ± 0.77	63.74 ± 4.45
Pleural effusion	75.95 0.68	70.66 ± 2.40	74.08 ± 2.24	63.76 ± 2.01

Classification balanced accuracies (%, ±SD) for CM (centralized model), LM (local model), GM (global model), and PM (personalized model) trained without data bias on CheXpert dataset for cardiomegaly and pleural effusion classes. The uncertainty is computed over three runs with different seeds and averaged over four datasets where applicable.

#### Biased setting

**Fig. 2:**
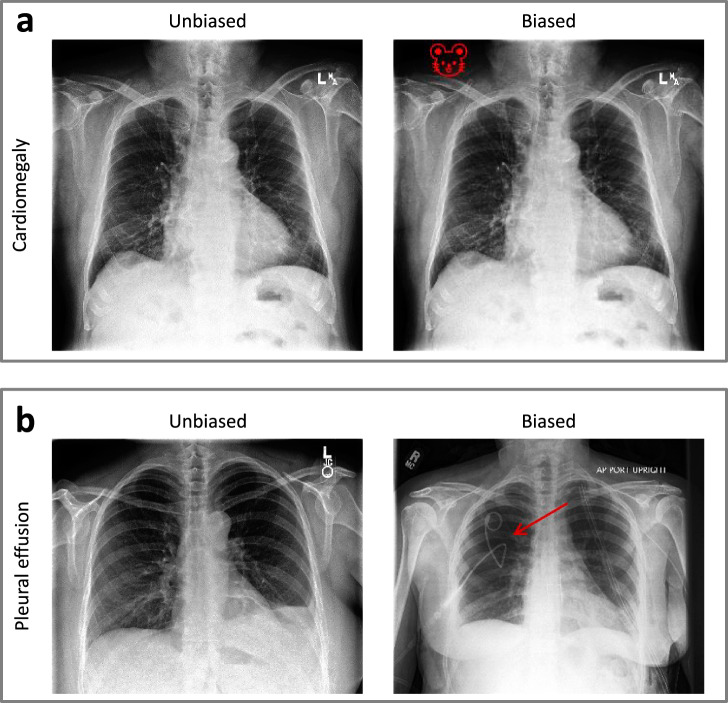
Examples of unbiased and biased (imperfectly interoperable) images from CheXpert dataset. For **a** cardiomegaly and **b** pleural effusion classes. The arrow indicates a chest drain.
- *real-world*: adding chest drains to a positive class in pleural effusion classification as a more real-world bias (Fig. 2b). To achieve this, we replaced images in a class Pleural effusion with X-rays labeled for the presence of chest drains^26^.

We experimented with two types of data bias in one of the clients:

- *synthetic*: adding a small red emoji to a positive class in cardiomegaly classification (Fig. 2a);

The real-world use case can arise as pleural effusions are often drained. Drain positions are routinely checked with a post-insertion X-ray. Thus, a model may learn to diagnose pleural effusion by detecting a chest drain, rather than the pathology (i.e., shortcut learning).

We compare local, global and presonalized models (LM^b^, GM^b^, and PM^b^, respectively) trained in the presence of data bias separately on unbiased and biased data (Table 2). It is clear that both types of data poisoning have a large effect on model performance.

**Table 2:** Effect of bias in FL

Model	LM^b^		GM^b^		PM^b^	
Test set	Biased	Unbiased	Biased	Unbiased	Biased	Unbiased
Cardiomegaly	100.0 ± 0.0	50.0 ± 0.0	61.53 ± 4.27	55.85 ± 3.69	89.80 ± 10.20	50.0 ± 0.0
Pleural effusion	73.22 ± 1.16	50.37 ± 0.38	49.72 ± 0.28	50.01 ± 0.01	64.81 ± 0.99	49.87 ± 0.10

Classification balanced accuracy (%, ± SD) for LM^b^, GM^b^, and PM^b^ trained in an FL setting with one biased client on the CheXpert dataset for cardiomegaly and pleural effusion classes. For each model, the value in the left subcolumn corresponds to the test set of a biased client, and in the right subcolumn, there is an average value over the test sets of unbiased clients. The uncertainty is computed over three runs with different seeds and averaged over four datasets where applicable.

We see that models with large local contributions, LM^b^ and PM^b^, give 100.0% and 89.80 % test accuracy, respectively, on biased data and 50.0% on unbiased ones in the case of cardiomegaly classification. Thus, these models strongly rely on the presence of bias to predict a positive class (shortcut learning).

Since the chest drain bias is more difficult to learn than the obvious emoji, for pleural effusion classification, LM^b^ and PM^b^ do not achieve maximum accuracy on biased data but instead 73.22% and 64.81%, respectively. At the same time, their performance on the unbiased test set is as low as for the cardiomegaly class, namely 50.37% and 49.87% for LM^b^ and PM^b^, respectively.

Global models (GM^b^), trained via communication of all the learnable parameters of ProtoPNet, demonstrate a performance different from their local and personalized versions. For cardiomegaly, GM^b^ achieves 61.53% and 55.85% balanced accuracy on biased and unbiased sets, respectively. For pleural effusion, the model achieves nearly 50% on both test sets. Sensitivity and specificity for the biased setting are shown in Supplementary Table 2.

### Qualitative results

**Fig. 3:**
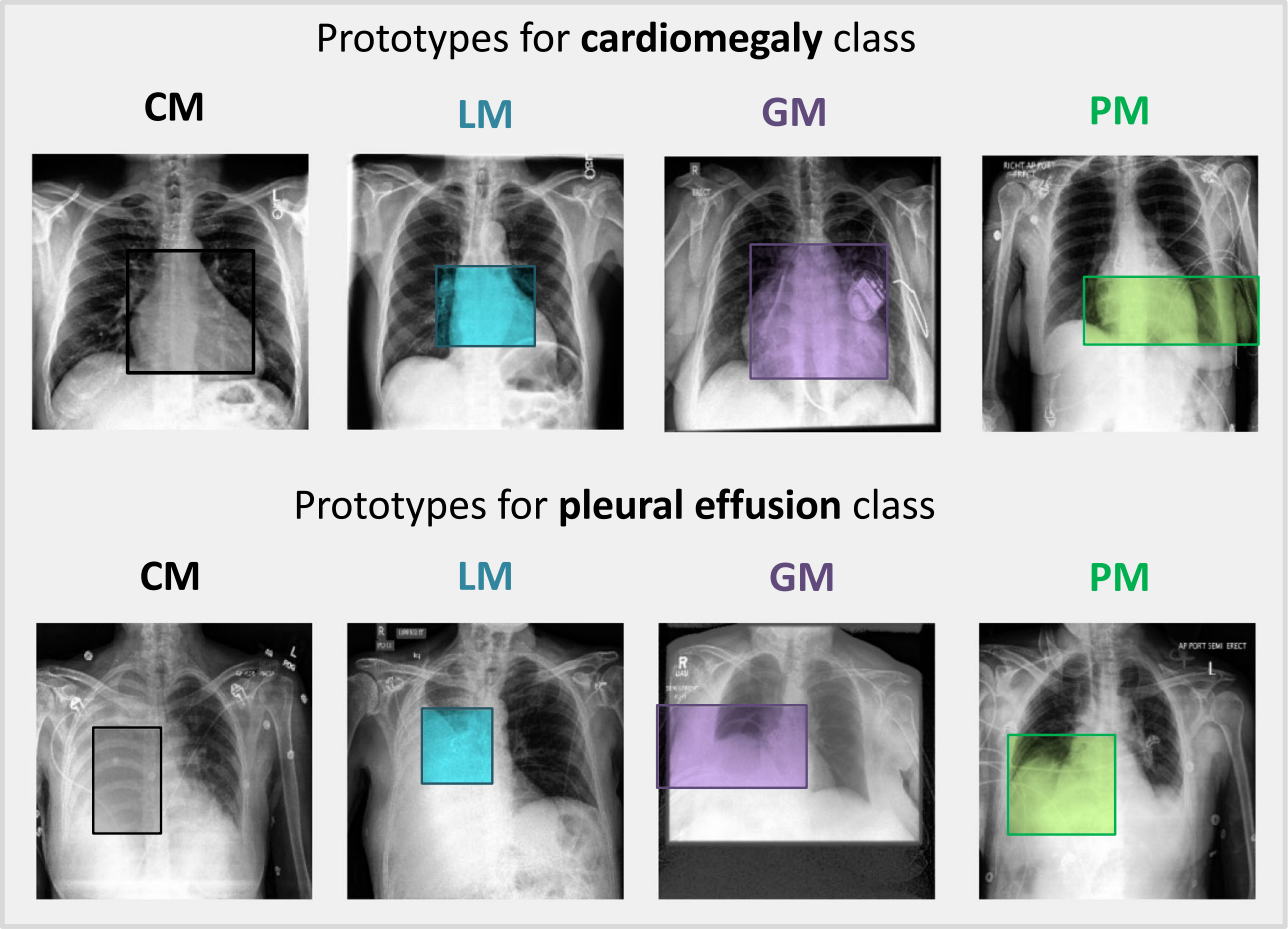
Prototypes learned in an unbiased setting. Examples of prototypical parts learned by CM (gray), LM (blue), GM (purple), and PM (green) in an unbiased setting and visualized on a corresponding training set.

The quantitative performance of the models described above can be further supported in a visually interpretable way with the help of learned prototypes. The examples of prototypes visualized on training sets for the models trained in the unbiased setting are shown in Fig. 3. We can see that these prototypes represent class characteristic features that align with human logic. For example, in order to classify an image as cardiomegaly, a centralized model looks at the whole enlarged heart (Fig. 3) or at the collarbone level in the center, pointing out the extended aorta characteristic for this condition (Supplementary Fig. 2). As for the pleural effusion classification, most prototypes activate the lower part of the lungs, where fluid accumulates in this disorder. More examples of the prototypes learned in the unbiased and biased settings can be found in Supplementary Figs. 2–15.

**Fig. 4:**
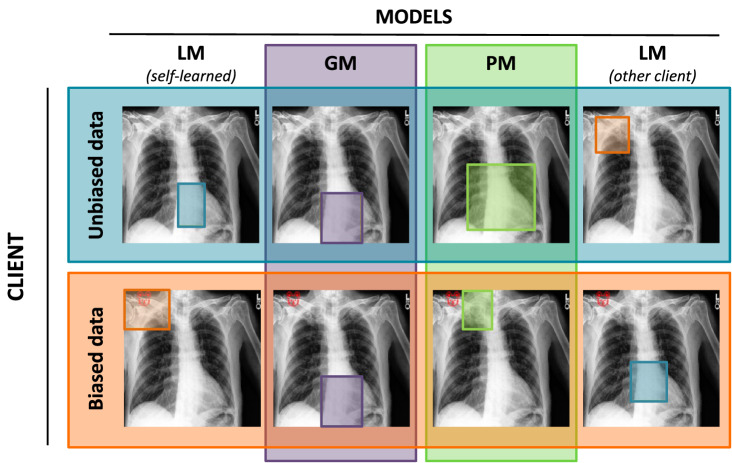
Bias identification in the cardiomegaly classification task with MyTH. Examples of a test image with bounding boxes indicating the most activated patches by the prototypes learned locally and globally on unbiased (blue) and biased (orange) CheXpert datasets in an FL setting for cardiomegaly classification. The difference between local (LM) and global (GM/PM) prototypes signals about poor data interoperability in the federation.

**Fig. 5:**
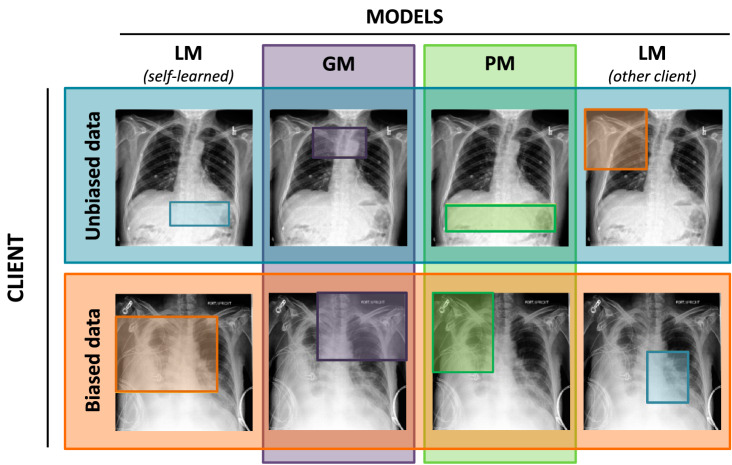
Bias identification in the pleural effusion classification task with MyTH. Examples of a test image with bounding boxes indicating the most activated patches by the prototypes learned locally and globally on unbiased (blue) and biased (orange) CheXpert datasets in an FL setting for pleural effusion classification. The difference between local (LM) and global (GM/PM) prototypes signals about poor data interoperability in the federation.

To demonstrate the effect of data bias, we compare the models on *test* images by finding a patch mostly activated by the prototypes learned locally and collaboratively in the FL setting with three unbiased and one biased client (Figs. 4 and 5). We see that the local model of an unbiased client *looks at* a meaningful class-characteristic patch in both biased (Figs. 4 and 5: second-row last column) and unbiased (Figs. 4 and 5: first-row first column) images to reason its predictions. The personalized model of an unbiased client highlights a meaningful patch too (Figs. 4 and 5: first-row third column). In the case of a biased client, the local model (LM^b^) for cardiomegaly classification (Fig. 4: second-row first column) *looks at* the emoji in the upper left corner of a test image. It tends to search for it in the unbiased image as well (Fig. 4: first-row last column). The neighborhood of this injected bias turned out to be the most activated patch for the personalized model (PM^b^, Fig. 4: second-row third column). This result explains the 100% accuracy of LM^b^ and 89.80% accuracy for PM^b^ on a biased test set and their complete failure on an unbiased one.

In the pleural effusion class, LM^b^ and PM^b^ indeed rely on the presence of a chest drain in an X-ray image, as we can see from the most activated prototypes (Fig. 5: second row first and third columns).

As for the fully global models trained in the federated setting with one biased client (GM^b^), there is a difference in their behavior depending on the type of bias used. Injected bias (an emoji), applied to the cardiomegaly class, did not have an effect on the global prototypes: they still activate the heart in both biased and unbiased images (Fig. 4: second column). For pleural effusion, however, with a more realistic chest drain bias, the global prototypes seem to be affected strongly by the biased client’s training set since they tend to activate the upper part of an image instead of the bottom of the lungs where the fluid usually accumulates in the pleural effusion condition (Fig. 5: second column).

We demonstrated that prototypes learned by ProtoPNet are sensitive to data bias and thus can help to create a visually interpretable approach to explore data interoperability in FL in a privacy-preserving way. We discuss this possibility in the next section.

## Discussion

Data compatibility between the clients in FL is of utmost importance for training an efficient and generalizable model. In this work, we present a visually interpretable approach for bias identification in FL that leverages a prototypical part learning network. A scheme to identify an incompatible client can be approximated as follows:

1. Each client trains a local ProtoPNet (LM) on its own data set.
2. With the help of a central server, the clients train global models (GM and/or PM), sharing all or a portion of learnable parameters (e.g., only the prototypes and weights of the final layer).
3. Each client visualizes its *local*, *global*, and *personalized* prototypes (finding the most activated patches) on its local test set and compares them by means of simple visual inspection (ideally with the help of domain experts). There is no need to share a test set with other clients or the server.
4. A large difference between local and global/personalized prototypes for certain clients indicates a possible data bias in the federation and requires the clients to either quit the federation or take measures to improve the quality of their training data.

We demonstrate this scheme on a task of binary classification of X-ray images for the presence of cardiomegaly and pleural effusion conditions using two different data poisoning patterns. As can be seen from Fig. 4, simple injected bias such as an emoji in the cardiomegaly class easily confuses the local model making it spuriously rely on this emoji to predict a positive class. It is interesting to note that for this binary classification task, adding bias to a positive class also changes the prototypes for a negative class. This effect can be seen in Supplementary Fig. 4 and Supplementary Fig. 8, where prototypical parts for a negative (unbiased) class turned out to be left in upper regions where there was an emoji for a positive class. Obviously, these prototypes have no practical value or plausible physiological mapping in classifying cardiomegaly.

Training a model via averaging the parameters over all clients helps to level out the effect of the bias completely (GM) or, to a smaller extent (PM). This apparent difference between local and global/personalized prototypes should alarm the data owner of possible discrepancies between their data and others. From the unbiased clients' perspective, since the difference between the prototypes for them is negligible, a drop in the performance of a GM in comparison to LM and larger uncertainty values are signs of poor data interoperability in the federation.

To experiment with more practically relevant data bias, we mimic a common real-world example of shortcut learning, where pleural effusion can be detected by the presence of chest drains (that have been placed after initial diagnosis as a therapeutic intervention). Thus the presence of chest drains in X-ray images can serve as a proxy for pleural effusion class. We trained our models in the FL setting where one client has images with chest drains in the positive class (note that these images do not necessarily have pleural effusion anymore).

Figure 5 shows a possible output of applying MyTH on a pleural effusion classification task in a biased setting. As before, an LM^b^ fails to activate a class-relevant feature, namely the bottom region of the lungs, as an unbiased model does and instead *looks at* the upper part of the chest where there are lots of drains. The same result was observed for PM^b^. It is interesting that the fully global model also activates the upper part of a test image in both biased and unbiased samples. Unlike the cardiomegaly classification, in this case, the data incompatibility is clearer for unbiased clients than for biased ones. Indeed, in the cardiomegaly classification task, only one client has a systematic bias, while in the pleural effusion case, chest drains may naturally be present in the images of other clients as well. This data distribution is applicable in the real world. It makes the chest drain prototype dominant among the positive class prototypes of a global model and significantly worsens the overall model performance.

As mentioned in the “Experimental details” subsection in “Methods” section, the two different ways of parameter aggregation allow us to investigate a trade-off between privacy and ease of bias identification. Obviously, the more parameters clients share, the higher the risk of privacy leakage. At the same time, GM^b^ trained via aggregating all learnable parameters of ProtoPNet demonstrates a large difference between local and global prototypes in case of the presence of data bias in the federation facilitating the identification of this bias. PM^b^, trained by centrally updating only the prototypes and weights of the final layer, makes it more challenging for an adversary to get the data from such a small set of network parameters but have less bias-identification power: due to a large local contribution, the difference between local and personalized prototypes is small.

In this work, we presented two extreme cases of parameter aggregation. More experiments are needed to define an optimal amount of parameters to share. Note, however, that this potential amount is not strict and depends on a certain data sensitivity to privacy. Therefore, it is up to clients to set their *privacy budget*, i.e. how many network parameters they are ready to share.

Optionally, clients can also share their *local* models with each other to visualize them on other clients’ data for additional comparison. An example of such a possibility is shown in Figs. 4 and 5 in the last column. We can see a large difference between the local prototypes learned on biased and unbiased data for both cardiomegaly and pleural effusion classes.

So far, we have been talking about data bias from a negative perspective. However, it is possible to have large heterogeneity among the clients meaning that some specific features that each of them has are important. For instance, variations in skin color can be an essential feature for predicting dermatological pathologies, as certain skin conditions may present differently depending on skin pigmentation. In this case, training PM allows clients to benefit from the federation while keeping their specific features essential for the prediction (see Figs. 4 and 5: second-row third column).

It is worth noting the directions for future work. To investigate the trade-off between privacy and bias-identification ability of our MyTH approach, further studies are required. It is also necessary to experiment with other medical datasets and real-world biased settings with a larger number of clients. Since the objective of this research was to develop a novel deep learning-based methodology with the potential for application across various medical imaging modalities, we used a retrospectively collected dataset to align with common practices in building deep learning models. However, a prospective study in a real clinical setting is strictly necessary to ensure the effectiveness and safety of our approach for practical use.

Additionally, a possible next step is to introduce a debiasing option to our approach that will allow us to instantly penalize the contribution of a biased client. It may be done, for example, automatically through prototypes weighing or manually with the help of domain experts.

Furthermore, we are currently working on adapting our MyTH approach to a web-based DISCO application (https://epfml.github.io/disco). It provides a user-friendly framework for distributed learning and thus has the potential to facilitate the integration of MyTH into medical practice.

Finally, a promising direction for future work is combining our approach with counterfactual explanation techniques known for providing human-understandable post-hoc explanations for model decision-making^27^. For instance, clients can apply counterfactual explanation techniques to their local and global models to observe the changes in model output after removing features identified by the MyTH approach as potential biases. This integration can further help in developing trust in deep learning models among medical practitioners.

## Methods

### Model description

The ProtoPNet architecture is presented in Supplementary Fig. 1. The network is composed of the following parts:

- a set of convolutional layers to learn features from the input data;
- two additional 1 × 1 convolutional layers with *D* channels and the ReLU activation after the first layer and Sigmoid after the second;
- a prototype layer with a predefined number of prototypes. Each prototype is a vector of size 1 × 1 × *D* with randomly initialized entries;
- a final fully connected layer with the number of input nodes equal to the number of prototypes and the number of output nodes corresponding to the number of classes. The weights indicate the importance of a particular prototype for a class. They are initialized as in ref. ^22^ such that the connection between the prototypes and their corresponding class is 1 and −0.5 for the connections with the wrong classes.

**Fig. 6:**
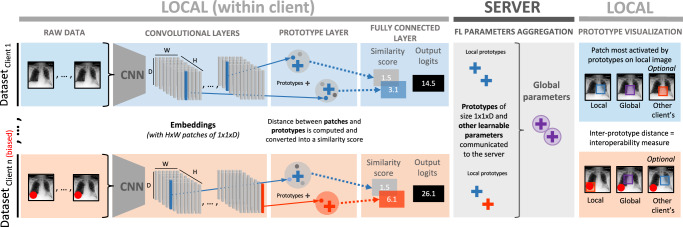
MyTH architecture. Several clients (blue panel for unbiased and orange panel for biased clients) wish to learn a model in a federated setting via a SERVER. MyTH passes raw data through convolutional layers to create embeddings in a latent space, each of which can be seen as *H* × *W* image patches of size [1 × 1 × *D*]. These patches are clustered around the closest prototypes (blue and red crosses), which are being learned for each class in the prototype layer. The prototype is a vector representing a class-characteristic feature in the latent space. *Client*_*n*_ (orange panel) has a systematic bias, which contaminates the prototype pool (red cross). Prototypes for each class and other learnable parameters of the network are shared with the SERVER by each client and aggregated to make global parameters (circled purple cross). These are then sent back to the clients. Classification is based on a similarity score between the prototypes and the patches of an encoded image. In the final panel, we see how global and local prototypes can be compared without sharing any original data.

We trained MyTH, an FL adaptation of ProtoPNet, using either unbiased identical data distribution among clients (unbiased setting) or imperfectly interoperable with systematic bias in a single class (biased setting). Two parameter aggregation schemes were applied: (1) a central server aggregates and updates local parameters of all the layers of ProtoPNet or (2) only the prototypes and weights of the final fully connected layer. In the second case, the parameters of feature learning layers always stay local, which results in *personalized* models with global prototypes. A detailed scheme is shown in Fig. 6.

By learning local prototypes, each client identifies the features in its training images most important for the task. In contrast, the global prototypes show the relevance for all clients on average. Finally, by examining the difference between local and global prototypes, a client can identify bias in its own or in another client’s dataset.

Experimenting with two different parameter aggregation schemes allows us to investigate the trade-off between the bias-revealing and privacy-preserving properties of MyTH.

#### Notation

Hereafter, we denote matrices and vectors in bold capital and bold lowercase letters, respectively.

*Data split*: Each client *n* ∈ {1, . . ., *N*} has a training set **D**^*n*^ of size *l*, which consists of training images ${\{{{\bf{X}}}_{i}\}}_{i = 1}^{l}$ and their corresponding classes ${\{{y}_{i}\}}_{i = 1}^{l}$.

*Model*: Each client learns features with convolutional layers and *m* local prototypes ${{\bf{P}}}^{n}={\{{{\bf{p}}}_{j}\}}_{j = 1}^{m}$ of size [1 × 1 × *D*] with a fixed number of prototypes per class. First, given an input image **X**_*i*_, the convolutional layers produce an image embedding **Z**_*i*_ of size [*H* × *W* × *D*], which can be represented as [*H* × *W*] patches of size [1 × 1 × *D*]. Then, the prototype layer computes the squared *L*^2^-distance between each prototype **p**_*j*_ and all the patches in the image embedding **Z**_*i*_. This results in *m* distance matrices of size [*H* × *W*] which are then converted into matrices of similarity scores (activation matrices) and subjected to a global max pooling operation to extract the best similarity score for each prototype. These final scores are then multiplied by the weight matrix ${{\bf{W}}}_{h}^{n}$ in the final fully connected layer *h* followed by softmax normalization to output class probabilities.

*Training*. The details of local training can be found in ref. ^22^ and in Supplementary Note 1. At the global update step, the server aggregates the local prototypes **P**^*n*^, weights of the final layer ${{\bf{W}}}_{h}^{n}$, and in the first aggregation scheme, also the parameters of the convolutional layers ${{\bf{W}}}_{c}^{n}$ from each client *n* and performs simple averaging of these parameters to obtain the global ones:

1 $${{\bf{P}}}_{{\rm {glob}}}=\frac{1}{N}\mathop{\sum }\limits_{n=1}^{N}{{\bf{P}}}^{n}$$

2 $${{\bf{W}}}_{h,{\rm {glob}}}=\frac{1}{N}\mathop{\sum }\limits_{n=1}^{N}{{\bf{W}}}_{h}^{n}$$

3 $${{\bf{W}}}_{c,{\rm {glob}}}=\frac{1}{N}\mathop{\sum }\limits_{n=1}^{N}{{\bf{W}}}_{c}^{n}$$

and then sends them back to clients as shown in Fig. 6.

To visualize the prototypes, each client finds for each of the local and global prototypes a patch among its training images from the same class that is mostly activated by the prototype. It is achieved by forwarding the image through the trained ProtoPNet and upsampling the activation matrix to the size of the input image. A prototype can be described as the smallest rectangular area within an input image that contains pixels with an activation value in the upsampled activation map equal to or greater than the 95th percentile of all activation values in that map^22^.

### Data

The experiments were conducted on the CheXpert dataset^25^, a large public dataset of 224,316 chest X-rays of 65,240 patients collected from Stanford Hospital between October 2002 and July 2017 in both inpatient and outpatient centers. Each image was accompanied by a radiology report which was labeled for the presence of 14 observations as positive, negative, or uncertain. The images were consequently labeled with a rule-based labeler developed by the authors of ref. ^25^, which extracted observations from the text radiology reports. The original 14 observations included Enlarged Cardiomediastinum, Cardiomegaly, Lung Lesion, Lung Opacity, Edema, Consolidation, Pneumonia, Atelectasis, Pneumothorax, Pleural Effusion, Pleural Other, Fracture, Support Devices, and No Finding. To simplify the experiments and interpretation, we used a *one-vs-rest* binary setting. Specifically, we use images with positive labels for classes Cardiomegaly or Pleural effusion as the positive class and all other images as the single negative class. Cardiomegaly is a health condition characterized by an enlarged heart, and pleural effusion is an accumulation of fluid between the visceral and parietal pleural membranes that line the lungs and chest cavity. This setting, however, resulted in a data imbalance (7 and 1.6 times for cardiomegaly and pleural effusion, respectively). To address this issue, we decreased the size of a negative class in the training set by undersampling to make it equal to the size of a positive class. The final training sets had 48,600 and 37,088 images for cardiomegaly and pleural effusion classification, respectively. The validation sets were left imbalanced.

### Experimental details

For both cardiomegaly and pleural effusion classification tasks, we first trained and evaluated a baseline centralized ProtoPNet, which we denote as Centralized Model (CM). Then we made an IID partition of the data over four clients and trained Local (LM), Global (GM), and Personalized (PM) models. Finally, we introduced systematic bias to one client’s dataset and trained LM^b^, GM^b^, and PM^b^ models where superscript b denotes a setting with one biased and three unbiased clients. The training details are described below.

#### Unbiased setting

1. Centralized (CM) ProtoPNet. As a baseline, we follow the architecture and optimization parameters from the ProtoPNet paper^22^ using the DenseNet^28^ convolutional layers pretrained on ImageNet^29^ to learn a CM on the whole dataset. We used 10 prototypes of size 1 × 1 × 128 per class. We report balanced average validation accuracy due to the validation set imbalance:
  4 $${\rm {Balanced}}\,{\rm {accuracy}}=\frac{{\rm {Sensitivity+Specificity}}}{2}$$
2. Local models (LM): We trained and evaluated LM for each of the four IID clients.
3. Global models (GM): Using the first FL setup where the server aggregates parameters of all the layers, GMs were trained according to the scheme depicted in Fig. 6. The training comprises three (for pleural effusion) or four (for cardiomegaly) communication rounds between the clients and the server. The server initializes a ProtoPNet model and sends it to the clients who learn LMs. After five epochs, a subset of local parameters is communicated to the server and aggregated. Importantly, during this training stage, each client keeps the pretrained convolutional weights frozen and trains two additional convolutional layers. Each of the next communication rounds includes the following steps:
  *Local training*: Each client trains convolutional layers, a prototype layer, and a final fully connected layer locally on its own dataset.
  *Local parameters*: A set of local prototypes **P**^*n*^, weights ${{\bf{W}}}_{h}^{n}$ and ${{\bf{W}}}_{c}^{n}$ is sent to the server after every 10 epochs.
  *Global parameters*: The server averages local parameters to create a set of global prototypes **P**_glob_, weights **W**_*h*,glob_ and **W**_*c*,glob_. These are shared back to each client to iterate training.
4. Personalized models (PM): We used the second FL setup within which the server aggregates only the prototypes **P**^*n*^ and weights of the final fully connected layer ${{\bf{W}}}_{{h}}^{n}$ to train PMs. We followed the same communication technique as described for GM, with the difference that, after receiving the updated prototypes **P**_glob_ and weights **W**_*h*,glob_ from the server, each client performs an additional prototype update locally by finding the nearest latent training patch from the same class and assigning it as a prototype. This operation is known as prototype *push* in ref. ^22^, and we use it to adapt global prototypes to a local dataset for personalization. This step is followed by local optimization of the final layer to improve accuracy.

#### Biased setting

**Fig. 7:**
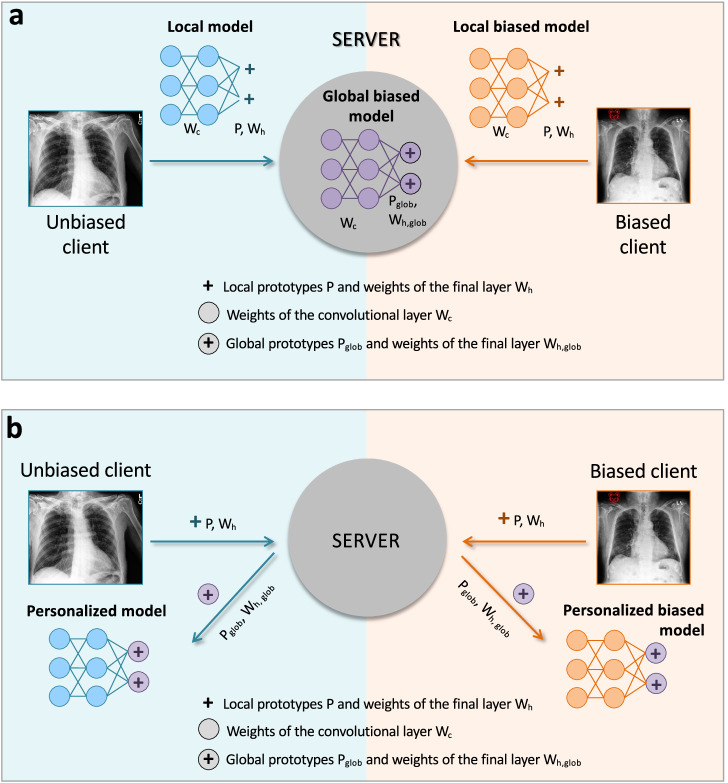
Schematic representation of the MyTH training in a biased setting. **a** For the global biased model (GM^b^), the clients send to the server parameters of the convolutional layers (*W*_*c*_, denoted by blue circles for unbiased client and orange circles for biased client), along with prototypes and weights of the final fully connected layer (*P* and W_*h*_, denoted jointly by blue and orange crosses), i.e. their entire local models (LM and LM^b^). **b** To train personalized models (PM and PM^b^), the server aggregates and updates only the prototypes and weights of the final layer and sends these global updates (*P*_glob_ and *W*_*h*,glob_, denoted jointly by circled purple crosses) back to clients to finalize the training of the PMs. In this case, the parameters of the convolutional layers always remain local.

5. *Local, global, and personalized model*s: We trained LM^b^, GM^b^, and PM^b^ models in an FL setting with three unbiased clients and one with systematic bias in one class (Fig. 2). This setting is schematically shown in Fig. 7 between two clients. We visually inspect the prototypes learned locally and globally to detect the differences between clients’ data without sharing them.

### Statistical analysis

For each of the models described above, we present the uncertainty in terms of standard deviation. It was computed over three runs with different seeds. For LM and PM, the final performance was also averaged over four datasets (clients).

## Supplementary information

Supplementary information

## Data Availability

The benchmark CheXpert dataset (CheXpert-v1.0-small) used in the current study is publicly available on the Kaggle platform at https://www.kaggle.com/datasets/ashery/chexpert. A list of CheXpert images labeled for the presence of chest drains, which was used to generate biased data in this study, was adopted from ref. ^26^ and is publicly available at https://github.com/EPFLiGHT/MyTH.

## Code availability

The code to reproduce our approach is available at https://github.com/EPFLiGHT/MyTH.
