# Supplementary material for: MyThisYourThat for interpretable identification of systematic bias in federated learning for biomedical images
Source: NPJ Digit Med. 2024 Sep 7;7:238. doi: 10.1038/s41746-024-01226-1 (PMC11379706; doi:10.1038/s41746-024-01226-1)
Supplement: Supplementary file 1 — Supplementary information [file 41746_2024_1226_MOESM1_ESM.pdf]

Supplementary information

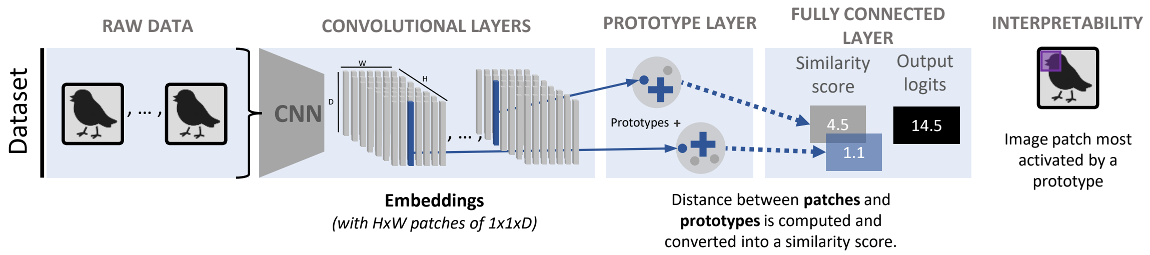

Supplementary Figure 1: `ProtoPNet` **architecture.** This is a centralized setting with no clients. `ProtoPNet` passes raw data through a CNN to create embeddings of size $[H \times W \times D]$ in the latent space, which can be seen as $H \times W$ image patches of size $[1 \times 1 \times D]$. These patches are clustered around the closest prototypes (blue crosses) which are being learned for each class in the prototype layer. The prototype is a vector representing a class-characteristic feature in the latent space. Classification is based on a similarity score between the prototypes and the patches of an encoded image. In the final panel, we see that the patch most activated by a certain prototype can be visualized directly.

Supplementary Note 1: **Local training description.** Given a set of training images $\mathbf{D}^n = \{(\mathbf{X}_i, y_i)\}_{i=1}^{l}$, where l is a number of images per client, each client aims to minimize the following objective:

$$\min_{\mathbf{P}^n, \mathbf{W}_c^n} \frac{1}{l} \sum_{i=1}^{l} \mathrm{CrsEnt}^n(h \circ g \circ f(\mathbf{X}_i), y_i) + \lambda_1 \mathrm{Clst}^n + \lambda_2 \mathrm{Sep}^n, \tag{1}$$

where f, g, and h denote the convolutional, prototype, and final fully connected layers, respectively. λ_1 and λ_2 are positive constants. CrsEnt is a cross-entropy loss that penalizes the misclassification, and the cluster and separation costs are defined as follows:

$$\mathrm{Clst}^n = \frac{1}{l} \sum_{i=1}^{l} \min_{j: \mathbf{p}_j^n \in \mathbf{P}_{y_i}^n} \min_{\mathbf{z}^n \in \mathrm{patches}(f(\mathbf{X}_i))} ||\mathbf{z}^n - \mathbf{p}_j^n||_2^2 \tag{2}$$

$$\mathrm{Sep}^n = -\frac{1}{l} \sum_{i=1}^{l} \min_{j: \mathbf{p}_j^n \notin \mathbf{P}_{y_i}^n} \min_{\mathbf{z}^n \in \mathrm{patches}(f(\mathbf{X}_i))} ||\mathbf{z}^n - \mathbf{p}_j^n||_2^2 \tag{3}$$

The minimization of the cluster cost (Clst) is needed to make each training image have a latent patch that is close to at least one prototype of the correct class. At the same time, every latent patch of a training image is separated from the prototypes of the incorrect classes through the minimization of the separation cost (Sep). More details about `ProtoPNet` can be found in [1] and in Supplementary Figure 1.

Supplementary Table 1: **Model performance in an unbiased setting.** Classification sensitivity and specificity for CM (centralized model), LM (local model), GM (global model), and PM (personalized model) trained without data bias on CheXpert dataset for cardiomegaly and pleural effusion classes. The uncertainty is computed over three runs with different seeds and averaged over four datasets where applicable.

Model	CM	LM	GM	PM
Cardiomegaly classification				
Sensitivity, $\pm$SD	0.66 $\pm$0.04	0.66 $\pm$0.04	0.68 $\pm$0.08	0.60 $\pm$0.08
Specificity, $\pm$SD	0.83 $\pm$0.02	0.77 $\pm$0.02	0.80 $\pm$0.06	0.67 $\pm$0.05
Pleural effusion classification				
Sensitivity, $\pm$SD	0.81 $\pm$0.02	0.69 $\pm$0.06	0.84 $\pm$0.08	0.69 $\pm$0.06
Specificity, $\pm$SD	0.71 $\pm$0.04	0.73 $\pm$0.04	0.64 $\pm$0.12	0.58 $\pm$0.02

Supplementary Table 2: **Model performance in a biased setting.** Classification sensitivity and specificity for LM^b, GM^b, and PM^b trained in an FL setting with one biased and three unbiased clients on the CheXpert dataset for cardiomegaly and pleural effusion classes. For each model, the value in the left subcolumn corresponds to the test set of a biased client, and in the right subcolumn, there is an average value over the test sets of unbiased clients. The uncertainty is computed over three runs with different seeds and averaged over four datasets where applicable.

Model	LM^b		GM^b		PM^b	
Test set	Biased	Unbiased	Biased	Unbiased	Biased	Unbiased
Cardiomegaly classification						
Sensitivity, $\pm$SD	1.0$\pm$0.0	0.0$\pm$0.0	0.46$\pm$0.28	0.34$\pm$0.30	0.80$\pm$0.20	0.0$\pm$0.0
Specificity, $\pm$SD	1.0$\pm$0.0	1.0$\pm$0.0	0.77$\pm$0.22	0.77$\pm$0.22	1.0$\pm$0.0	1.0$\pm$0.0
Pleural effusion classification						
Sensitivity, $\pm$SD	0.51$\pm$0.01	0.05 $\pm$0.02	0.0$\pm$0.0	0.0$\pm$0.0	0.37$\pm$0.03	0.07 $\pm$0.02
Specificity, $\pm$SD	0.96$\pm$0.01	0.96 $\pm$0.01	0.99$\pm$0.01	1.0$\pm$0.0	0.93$\pm$0.04	0.92 $\pm$0.02

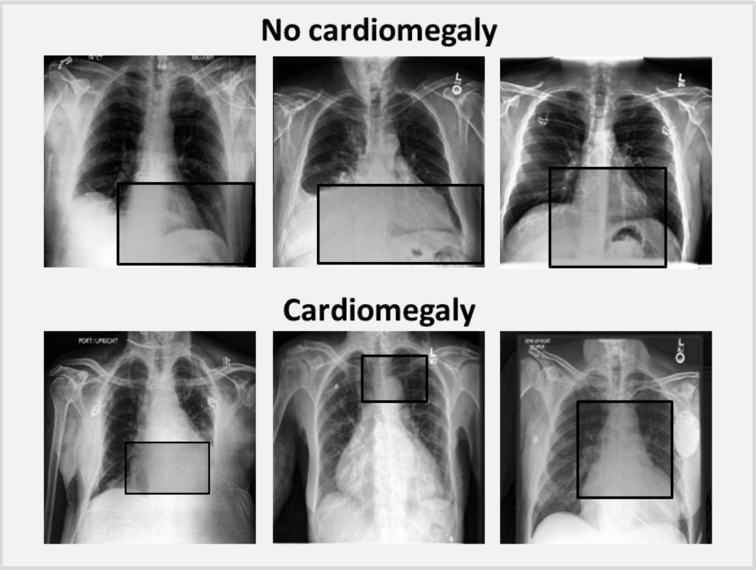

Supplementary Figure 2: **Centralized prototypes.** Examples of training images with bounding boxes indicating centralized prototypes learned on **unbiased** CheXpert data for *cardiomegaly* classification.

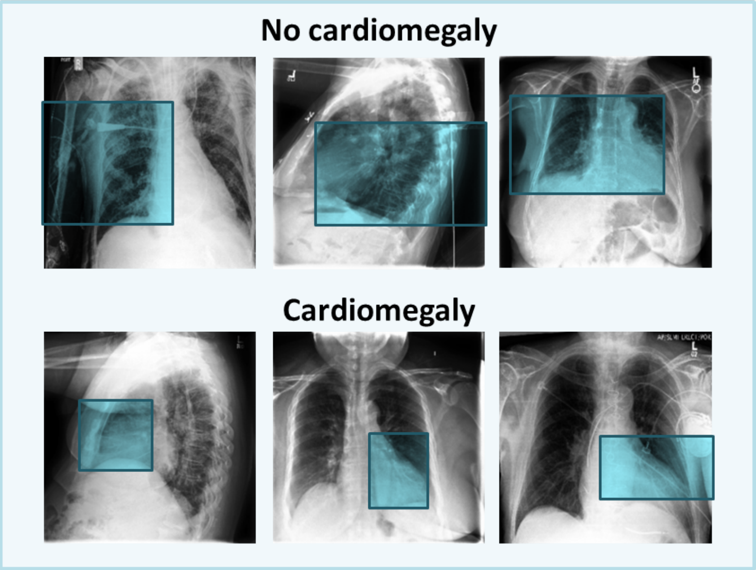

Supplementary Figure 3: **Local unbiased prototypes.** Examples of training images with bounding boxes indicating local prototypes learned on **unbiased** CheXpert data for *cardiomegaly* classification.

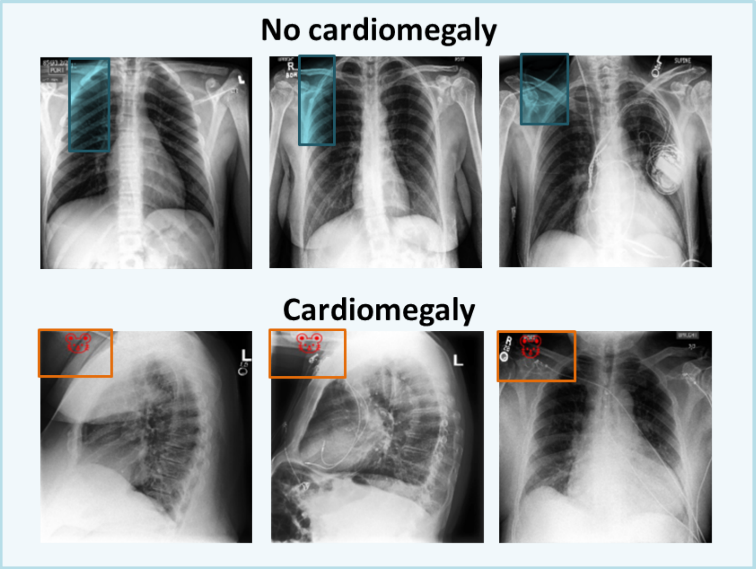

Supplementary Figure 4: **Local biased prototypes.** Examples of training images with bounding boxes indicating local prototypes learned on **biased** CheXpert data for *cardiomegaly* classification.

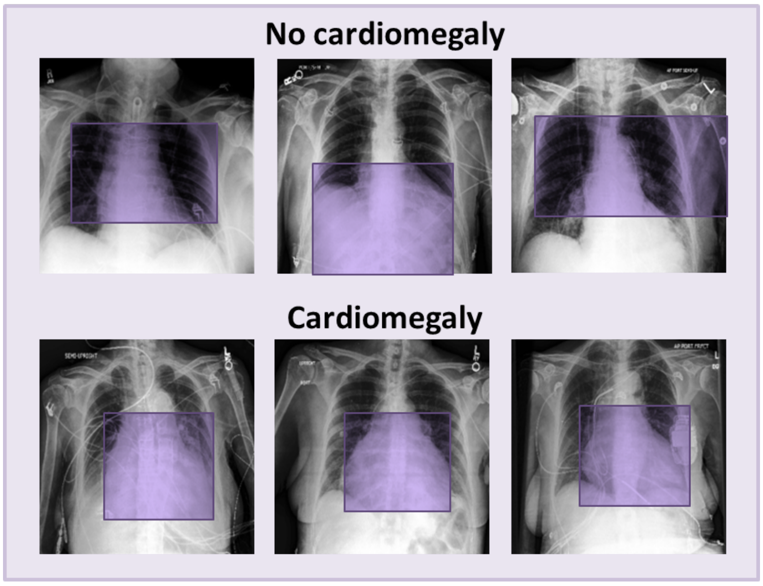

Supplementary Figure 5: **Global unbiased prototypes.** Examples of training images with bounding boxes indicating global prototypes learned on **unbiased** CheXpert data for *cardiomegaly* classification.

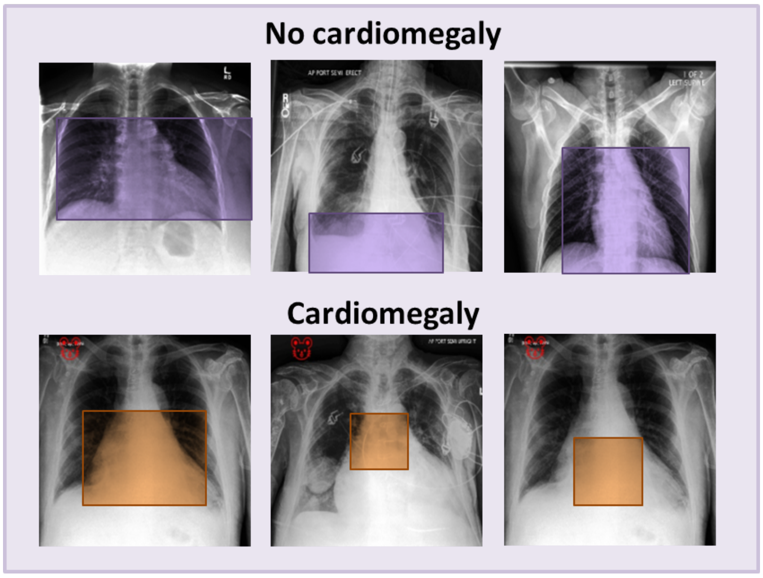

Supplementary Figure 6: **Global biased prototypes.** Examples of training images with bounding boxes indicating global prototypes learned on **biased** CheXpert data for *cardiomegaly* classification. The visualization is presented for the biased client.

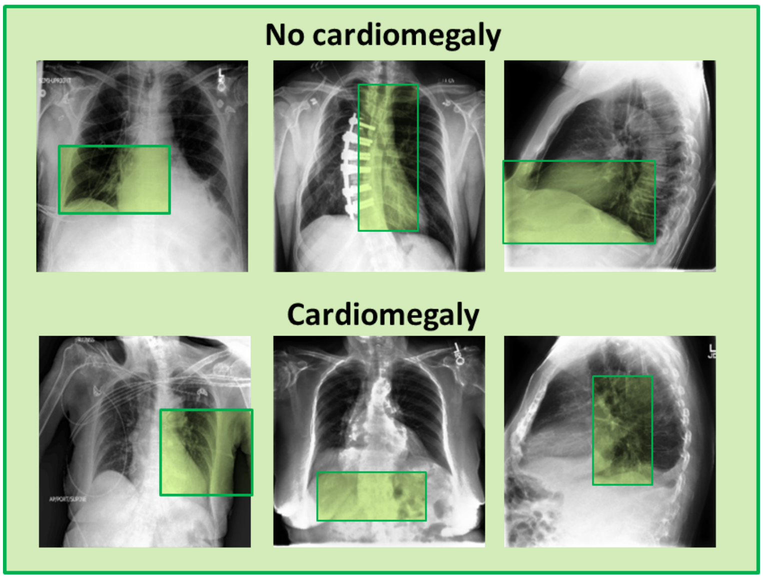

Supplementary Figure 7: **Personalized unbiased prototypes.** Examples of training images with bounding boxes indicating personalized prototypes learned on **unbiased** CheXpert data for *cardiomegaly* classification.

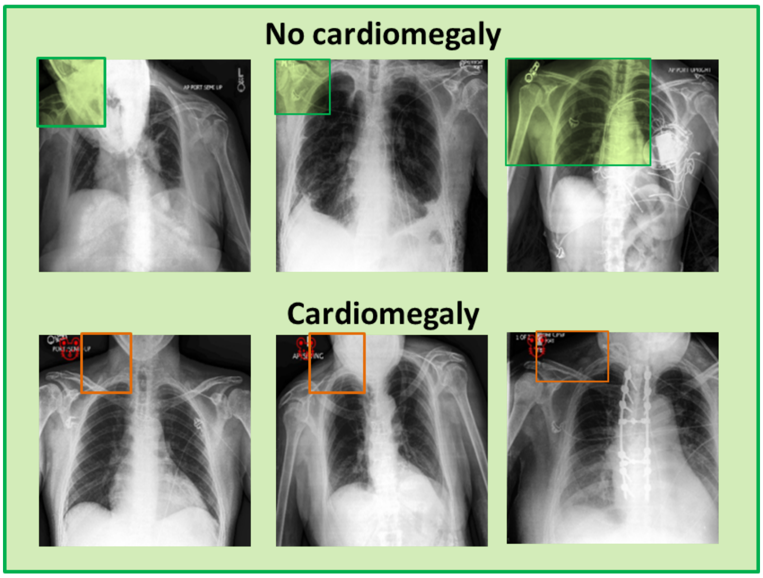

Supplementary Figure 8: **Personalized biased prototypes.** Examples of training images with bounding boxes indicating personalized prototypes learned on **biased** CheXpert data for *cardiomegaly* classification. The visualization is presented for the biased client.

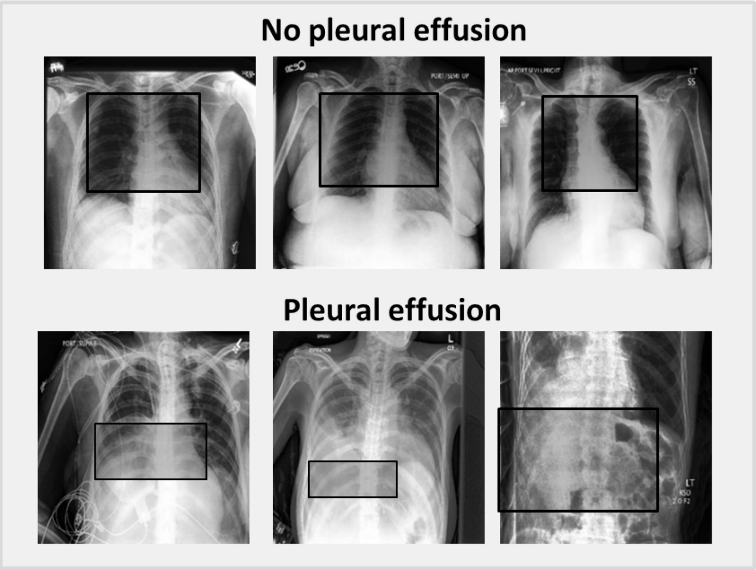

Supplementary Figure 9: **Centralized prototypes.** Examples of training images with bounding boxes indicating centralized prototypes learned on **unbiased** CheXpert data for *pleural effusion* classification.

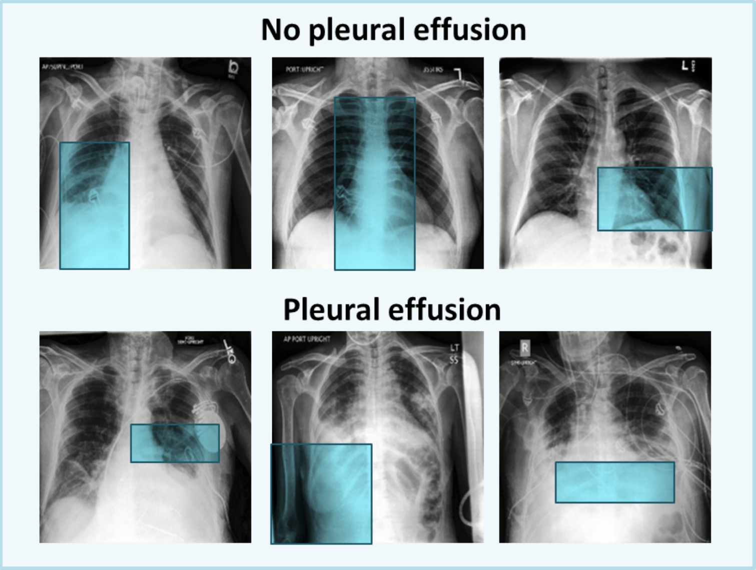

Supplementary Figure 10: **Local unbiased prototypes.** Examples of training images with bounding boxes indicating local prototypes learned on **unbiased** CheXpert data for *pleural effusion* classification.

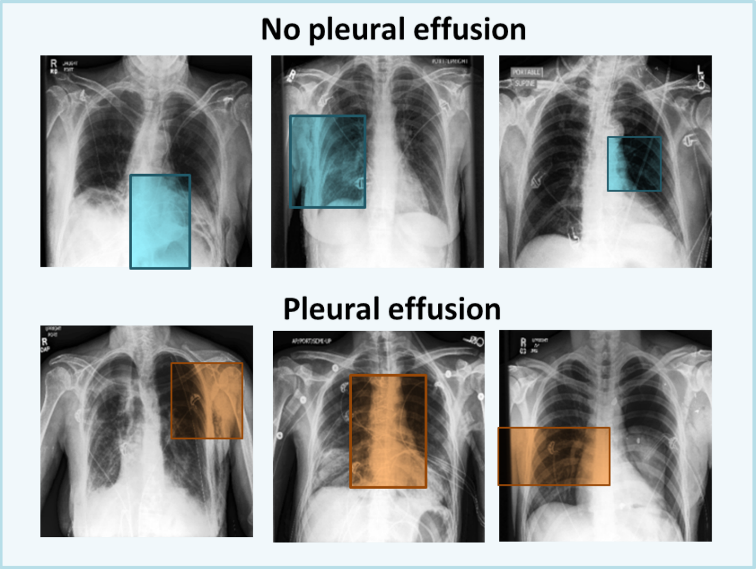

Supplementary Figure 11: **Local biased prototypes.** Examples of training images with bounding boxes indicating local prototypes learned on **biased** CheXpert data for *pleural effusion* classification.

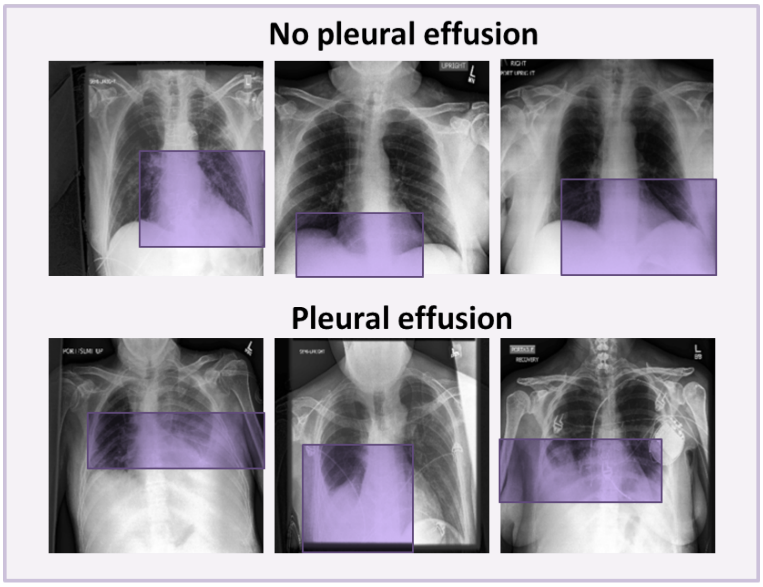

Supplementary Figure 12: **Global unbiased prototypes.** Examples of training images with bounding boxes indicating global prototypes learned on **unbiased** CheXpert data for *pleural effusion* classification.

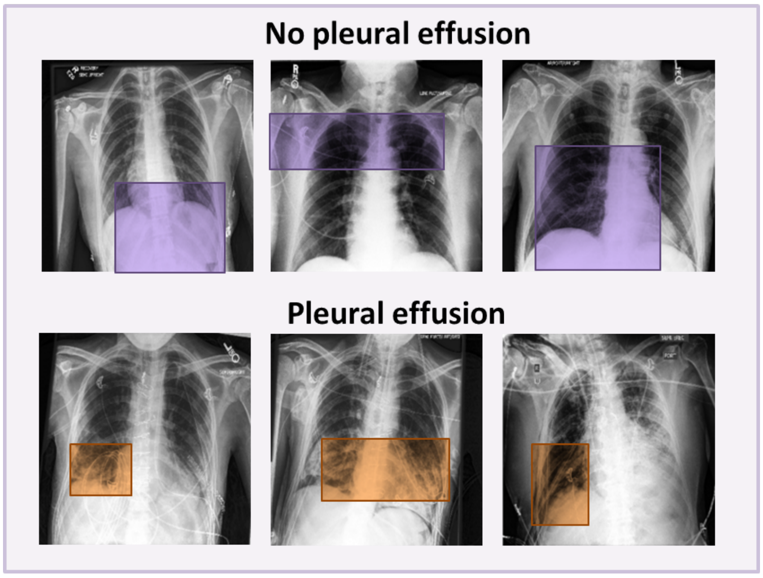

Supplementary Figure 13: **Global biased prototypes.** Examples of training images with bounding boxes indicating global prototypes learned on **biased** CheXpert data for *pleural effusion* classification. The visualization is presented for the biased client.

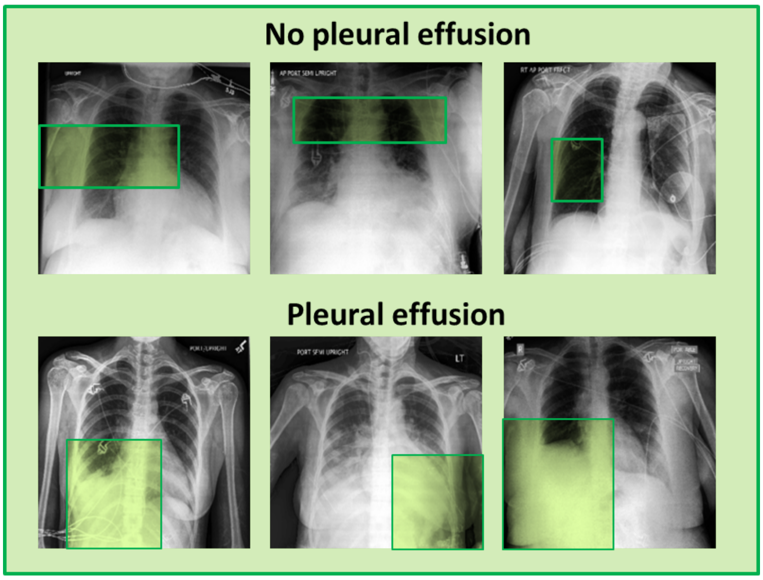

Supplementary Figure 14: **Personalized unbiased prototypes.** Examples of training images with bounding boxes indicating personalized prototypes learned on **unbiased** CheXpert data for *pleural effusion* classification.

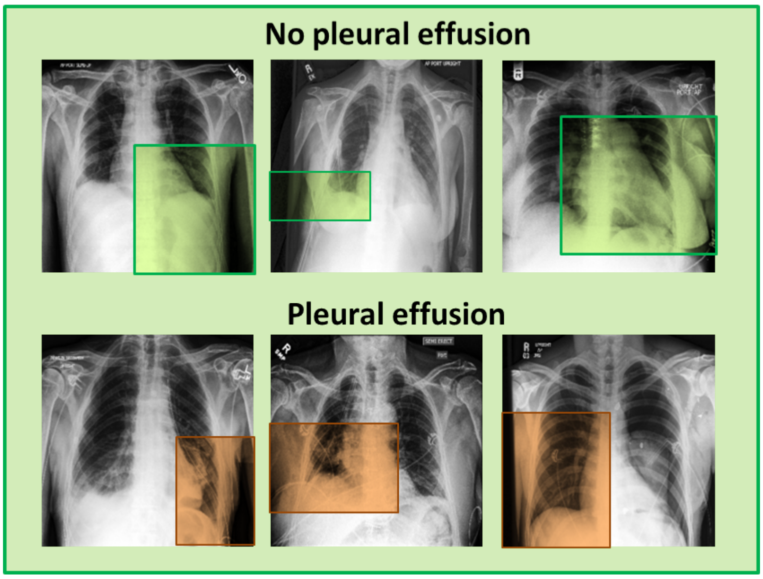

Supplementary Figure 15: **Personalized biased prototypes.** Examples of training images with bounding boxes indicating personalized prototypes learned on **biased** CheXpert data for *pleural effusion* classification. The visualization is presented for the biased client.

Supplementary References

1. Chen, C. *et al.* This Looks like That: Deep Learning for Interpretable Image Recognition. *Proceedings of the 33rd International Conference on Neural Information Processing Systems*, 8930–8941 (2019).
